# Methylene Blue Metabolic Therapy Restrains In Vivo Ovarian Tumor Growth

**DOI:** 10.3390/cancers16020355

**Published:** 2024-01-13

**Authors:** Jorgelindo da Veiga Moreira, Nancy Nleme, Laurent Schwartz, Kim Leclerc-Desaulniers, Euridice Carmona, Anne-Marie Mes-Masson, Mario Jolicoeur

**Affiliations:** 1 Research Laboratory in Applied Metabolic Engineering, Department of Chemical Engineering, Polytechnique Montréal, Montreal, QC H3T 1J4, Canada; 2 Assistance Publique des Hôpitaux de Paris, 75010 Paris, France; 3 Institut du Cancer de Montréal (ICM), Centre de Recherche du Centre Hospitalier de l’Université de Montréal (CRCHUM), Montreal, QC H2X 0A9, Canadaanne-marie.mes-masson@umontreal.ca (A.-M.M.-M.); 4 Department of Medicine, Université de Montréal, Montreal, QC H3T 1J4, Canada

**Keywords:** ovarian cancer, methylene blue, metabolic therapy, redox potential, mitochondria

## Abstract

**Simple Summary:**

This study confirms the potential of methylene blue (MB) in treatment strategies for ovarian cancer. Ovarian cancer cells taken from patients were inserted into mice, and their growth was followed together with mouse health. Our findings revealed MB’s ability to impede ovarian cancer progression, especially against tumors showing resistance to conventional chemotherapies. By investigating the effects of MB on cancer cell behavior and metabolism, this research revealed novel therapeutic targets for affecting tumor growth. These insights suggest that MB could be a valuable complementary therapeutic to existing treatments or an alternative for patients facing limited options. This research thus opens new avenues for developing targeted therapies that can remotely control cancer cell development.

**Abstract:**

Ovarian cancer remains a significant challenge, especially in platinum-resistant cases where treatment options are limited. In this study, we investigated the potential of methylene blue (MB) as a metabolic therapy and complementary treatment approach for ovarian cancer. Our findings demonstrated a significant in vivo reduction in the proliferation of TOV112D-based ovarian-cell-line xenografts. In this preclinical study, which used a carboplatin-resistant ovarian cancer tumor model implanted into mice, MB-mediated metabolic therapy exhibited superior tumor slowdown compared to carboplatin treatment alone. This indicates, for the first time, MB’s potential as an alternative or adjuvant treatment, especially for resistant cases. Our in vitro study on TOV112D and ARPE-19 sheds light on the impact of such an MB-based metabolic therapy on mitochondrial energetics (respiration and membrane potential). MB showed a modulatory role in the oxygen consumption rate and the mitochondrial membrane potential. These results revealed, for the first time, that MB specifically targets TOV112D mitochondria and probably induces cell apoptosis. The differential response of normal (ARPE-19) and cancer (TOV112D) cells to the MB treatment suggests potential alterations in cancer cell mitochondria, opening avenues for therapeutic approaches that target the mitochondria. Overall, our findings suggest the efficacy of MB as a possible treatment for ovarian cancer and provide valuable insights into the mechanisms underlying the efficacy of methylene blue metabolic therapy in ovarian cancer treatment.

## 1. Introduction

Ovarian cancer is a devastating disease, with the highest fatality rate among gynecological cancers [1]. Despite advances in cancer detection and treatment, the majority of patients with epithelial ovarian cancer (EOC) are diagnosed at an advanced stage and exhibit a limited response to platinum-based chemotherapy [2]. The development of drug resistance in advanced ovarian cancer often leads to relapse and death [3]. The current standard treatment options for platinum-resistant ovarian cancer, such as pegylated liposomal doxorubicin, paclitaxel, and topotecan, have shown limited efficacy and are accompanied by strong side effects [4]. Therefore, there is an urgent need to propose alternative therapeutic strategies to improve the outcomes for these patients.

Metabolic reprogramming has emerged as a key characteristic and hallmark of cancer cells, including ovarian cancer, contributing to their survival and growth [5,6,7,8]. An impaired mitochondrial function and an altered cellular metabolism play crucial roles in tumor progression and chemoresistance [9,10,11]. Mitochondria, the cellular powerhouses responsible for energy production and a major anabolic route, undergo profound alterations in many types of cancer cells (but remain active), promoting glycolysis and providing a selective advantage for tumor growth and survival [12,13,14,15,16]. 

One of the main intriguing metabolic phenomena associated with chemoresistance in ovarian cancer is the “Warburg effect” and enhanced glutaminolysis [17,18,19]. The Warburg effect is characterized by increased glucose consumption and lactate production, while enhanced glutaminolysis involves the increased utilization of glutamine as a fuel source for mitochondrial respiration and biosynthetic processes [17,18,19,20]. These metabolic alterations are commonly observed in various cancer cells, but are particularly prominent in chemoresistant ovarian cancer cells. In recent years, researchers have focused on understanding the metabolic aspects of cancer cells, with a specific emphasis on the role of mitochondria [21,22]. Otto Warburg’s ground-breaking work proposed that cancer is a metabolic disease caused by mitochondrial dysfunction, leading to increased anabolism and decreased catabolism. Expanding on these concepts, recent studies have highlighted the significance of mitochondrial energetics in cancer cells and have suggested that stimulating mitochondrial activity could be a potential strategy for overcoming chemoresistance. 

Methylene blue (MB), a well-known dye agent with antibacterial effects, has shown promising effects in stimulating the mitochondrial electron transfer chain and oxidative phosphorylation (OXPHOS), leading to increased mitochondrial respiration and ATP production [23,24]. It has been widely used as a strong photosensitizer for photodynamic therapy in cancer therapy. MB has been combined with gold nanoparticles (AuNPs) to enhance singlet oxygen generation without causing dark toxicity [25]. The treatment using these Au-polymer/MB-Tf nanoparticles showed a twofold increase in PDT efficiency compared to free MB. When applied to breast cancer, the results revealed an enhanced cytotoxicity in cells treated with the nanoparticles compared to MB alone, demonstrating an improved treatment efficiency [26]. MB has also been reported to have direct effects on cell and mitochondrial metabolism. Notably, studies have reported a strong decrease in lactate production and an improved mitochondrial capacity in cancer cells treated with MB [24,27]. These findings suggest that targeting the mitochondrial function using MB may offer a potential therapeutic approach for ovarian cancer treatment. 

In this study, we evaluated the effect of MB and that of METABLOC (a mixed solution of lipoic acid + hydroxycitrate) on TOV112D ovarian cancer cell viability as well as on mitochondrial energetics [28]. The in vitro studies were carried out on the TOV112D cell line and the normal ARPE-19 model to understand the differences in mitochondrial energetics, specifically cellular respiration, under the effect of MB and drug combinations (MB + METABLOC and MB + carboplatin). Moreover, the short-term effects of the metabolic therapies (MB and METABLOC) were evaluated using an in vitro TOV112D cell line model. A preclinical study was conducted to investigate the impact of metabolic therapies on ovarian-cancer-derived implanted tumors in mice. The main results showed that methylene blue, used as a metabolic therapy, can restrain in vivo ovarian tumor growth in mice. Using JC-1 as a mitochondrial membrane potential marker, we showed, for the first time, that MB may specifically induce cancer cell apoptosis. This is even more promising, as MB shows no apparent side effects in mice.

## 2. Materials and Methods

### 2.1. Statistical Analysis

The data are presented as the mean ± the standard error of the mean (*n* = 3). Statistical analyses were performed with GraphPad (Prism 9) and Excel. Values of *p* ≤ 0.05 were considered significant, and the notations of * (*p* ≤ 0.05), ** (*p* ≤ 0.01), and *** (*p* ≤ 0.001) were used for comparisons versus the control group using Student’s *t*-test.

### 2.2. Cell Lines and Culture Medium

The epithelial ovarian cancer cell line TOV112D (Cellosaurus, IS: CVL_3612), derived from patient cancerous tissue, was utilized in this study with the explicit permission of the CHUM (Centre Hospitalier de l’Université de Montréal) research ethics board (BD 04.002) [29]. The normal retinal epithelial cell line, ARPE-19, was procured from ATCC (CRL-2302) and employed as a suitable control model. TOV112D exhibited resistance to a carboplatin treatment in 2D (IC_50_ = 13.4 μM, based on a clonogenic assay) cultures [30]. Both cell lines were cultured in a complete OSE medium (Wisent, Cat. 316-030-CL, QC) supplemented with 10% fetal bovine serum (Wisent, Cat. 080-150, QC), 250 µg/mL of amphotericin B (Wisent, Cat. 450-105-QL, QC), and 50 mg/mL of gentamicin (Wisent, Cat. 450-135-XL, QC). Prior to conducting the experiments, the cells were incubated at 37 °C under a controlled atmosphere consisting of 21% O_2_ and 5% CO_2_. 

### 2.3. Xenograft Mouse Model

All animal treatments were carried out in compliance with the UK’s ARRIVE standards, the CRCHUM’s guidelines for the care and use of laboratory animals, and the recommendations of the CCAC (Canadian Council of Animal Care). The Institutional Animal Protection Committee (animal ethics committee), protocol number C22040AMMs, authorized this investigation. NOD.Cg-Rag1^tm1Mom^Il2rg^tm1Wjl^/SzJ immunodeficient female mice (007799, The Jackson Laboratory-JAX, Bar-harbor, ME, USA) were used for the xenograft with the TOV112D cell line. The mice were between the ages of 60 and 100 days at the start of treatment. A 200 μL suspension of 1 × 10^6^ TOV112D cells in 100 μL of cold Dulbecco’s PBS (311–425-CL, Wisent) with 100 μL of Matrigel^®^ matrix (CACB356237, Corning Inc., Corning, NY, USA) was injected subcutaneously into the flank of each mouse. Eight mice were employed for each of the vehicle control groups: the MB group, the MB + METABLOC group, the MB + carboplatin group, and the carboplatin group. Once the tumor reached 200 mm^3^ in size, the treatment started. Anti-nausea drugs (1 mg/kg of maropitant and 0.8 mg/kg of ondansetron) were administered one hour before the chemotherapy dosage to lessen the recognized adverse effects of carboplatin therapy. A total of 50 mg/kg/day of methylene blue (MB) was administered (in drinking water). METABLOC was administered through an intraperitoneal (IP) injection 3 times per week at a dose of 5 mg/kg of lipoid acid and 125 mg/kg of hydroxycitrate. Additionally, 50 mg/kg of carboplatin was IP-injected once per week. Using an intraperitoneal injection of euthanyl (pentobarbital sodium) at a dosage of 400 mg/kg (concentration of 240 mg/mL), the mice were slaughtered at the conclusion of the treatment period or if the ethical standards were met.

### 2.4. In Vitro Cell Proliferation Assay under the Methylene Blue, METABLOC, and Carboplatin Treatments

Among the tested concentrations, regarding methylene blue (Chimie-Plus Laboratoires, CAT. 81046, Saint Paul de Varax, France), the 50 μM MB concentration (MB-50), initially diluted from a liquid stock solution, elicited a 50% viability response in TOV112D. Hence, the MB-50 concentration was selected for all subsequent experiments. In the case of the carboplatin (Sigma, Cat. C2538, Oakville, ON, Canada) assays, a fixed concentration of 50 μM (CPN-50) was deliberately chosen to enable a comparison with the effects observed under the MB-50 condition. METABLOC refers to the combination of two molecules, lipoic acid and hydroxycitrate, utilized in this study. Lipoic acid was administered at a final concentration of 13 μM, while hydroxycitrate was used at a concentration of 125 μM. These specific concentrations were carefully chosen to explore the potential synergistic effects of these compounds in our metabolic therapy approach. Lipoic acid and hydroxycitrate have shown promising properties in previous research [28,31], making them valuable components of the METABLOC^®^ combination for investigating their impact on the metabolism and proliferation of ovarian cancer cells. Briefly, to ensure optimal adhesion to the plates, the cells were incubated overnight under normal growth conditions prior to initiating the experiments. Subsequently, the growth medium was replaced with MB-50, METABLOC, CPN-50, or the combination conditions. The cells were further incubated for 24 h before conducting the cell proliferation, qPCR, and mitochondrial membrane potential assays.

### 2.5. Flow Cytometry Analysis

We performed a flow cytometry analysis (BD Biosciences, Mississauga, Canada) as a marker of cell proliferation to mitigate the potential calorimetric interference caused by methylene blue (Laboratoire Chimie-plus, Saint Paul de Varax, France) that would affect standard metabolic assays. The viability was determined by normalizing the number of events to Precision Count Beads™ (BioLegend, Cat. 424902, San Diego, CA, USA) in the cell suspension. Similarly, the population of apoptotic cells was evaluated using Apotracker™ Green (BioLegend, Cat. 427402, San Diego, CA, USA) and normalized to the total cell population. Flow cytometric staining was performed by incubating 300 nM Apotracker™ Green per million cells in a final volume of 100 µL of PBS. Detection was carried out using the Alexa Fluor^®^ 488 channel. The mitochondrial membrane potential was evaluated in the TOV112D cancer line and the normal ARPE-19 cell line using the JC-1 probe (ThermoFisher Scientific, Cat. T3168, Burlington, ON, Canada) and flow cytometry. The cells were subjected to metabolic treatments for 4 h. JC-1 exhibited potential-dependent accumulation in mitochondria, shifting the fluorescence emission from green (~525 nm) to red (~590 nm). The polarized mitochondria displayed red fluorescence due to aggregate formation, while the depolarized mitochondria exhibited green fluorescence as monomers. All the flow cytometry data were analyzed using the FlowJo software 10.9 (BD Biosciences, Mississauga, ON, Canada).

### 2.6. Oxygen Consumption Assay

The continuous monitoring of the oxygen consumption rates was conducted in the two cell lines under various conditions (MB-50, METABLOC, and carboplatin-50 treatments) using the Resipher 32× device (Lucid Scientific, Atlanta, GA, USA). The oxygen sensor was immersed in 100 µL of the cell culture within specially adapted 96-well Falcon plates (Corning, Cat. 353072, Santa Barbara, CA, USA). The oxygen consumption rate (OCR) measurements were initiated after 5 h, considering that the oxygen in the medium had reached saturation on the surface. The OCR measurements captured the dynamic exchange of oxygen between the medium and cellular consumption. To evaluate the impact of the different drugs on cell respiration, the drugs were added after 24 h of incubation, allowing for an assessment of their effects on oxygen consumption.

### 2.7. Quantitative PCR for Respiratory mRNA Complex Estimation

The ARPE-19 and TOV112D cell lines were subjected to the MB-50, METABLOC, and carboplatin-50 treatments for 24 h, and then the total RNA was extracted using RNA extraction kits (Qiagen, Cat. 74004, Toronto, ON, Canada). The total RNA underwent a DNase treatment and was then reverse-transcribed using the Maxima First Strand cDNA synthesis kit, which included ds DNase (ThermoFisher Scientific, Cat. K1641). We assessed the gene expression through assays crafted via the Universal Probe Library (Roche, Cat. UPL1THRU10, Laval, QC, Canada). A standard curve was established for every qPCR assay to confirm that its efficiency ranged from 90% to 110%. The QuantStudio qPCR device (ThermoFisher Scientific) was employed to measure the amplification rate. To determine the relative expression (RQ = 2^−ΔΔCT^), we utilized the Expression Suite software v1.2 (ThermoFisher Scientific, Burlington, Canada), taking both GAPDH and B2M as internal controls.

## 3. Results

### 3.1. Metabolic Therapy Effects on Cell Proliferation

**Figure 1:**
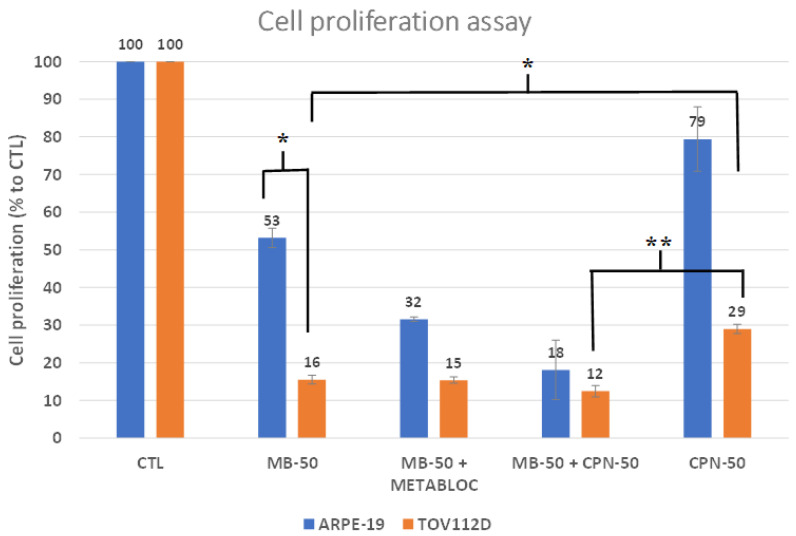
Cell proliferation under metabolic treatment. Methylene blue metabolic therapy strongly inhibited TOV112D cancer cell line proliferation. Proliferation of the normal cell line ARPE-19 and the ovarian cancer line TOV112D was assessed following methylene blue metabolic therapy at 50 μM (MB-50) or in combination with METABLOC (MB-50 + METABLOC). Carboplatin at 50 μM (CPN-50) was included for comparison. The proliferation assay was conducted for 24 h. The TOV112D cancer cell line exhibited a high sensitivity to MB-50 compared to ARPE-19, with proliferation reaching only 16% of the control compared to 53% for ARPE-19. Addition of METABLOC to MB-50 had no additional effect on the cancer cell line, while it moderately affected ARPE-19 (32%). The addition of carboplatin to MB-50 slightly impacted TOV112D proliferation compared to MB-50 alone, while it displayed increased resistance to CPN-50 treatment alone. CPN-50 only marginally affected ARPE-19 proliferation (79% of control). The significant notations are * (*p* ≤ 0.05) and ** (*p* ≤ 0.01).

Methylene blue metabolic therapy exerted a strong inhibitory effect on the proliferation of the TOV112D ovarian cancer cell line (Figure 1), where the cell proliferation reached 16% of the control in response to the MB-50 treatment. In contrast, the ARPE-19 cells showed a significantly higher proliferation rate, reaching 53% of the control under the MB-50 condition. The sensitivity of the TOV112D cells to MB-50 compared to ARPE-19 (*p* ≤ 0.05) highlights the potential of methylene blue as a therapeutic agent for targeting ovarian cancer cell metabolism. It is also noteworthy that MB-50 had a significantly greater inhibitory effect on the proliferation of TOV112D compared to CPN-50—16% proliferation against 29%, respectively (*p* > 0.05). Interestingly, the addition of METABLOC to MB-50 did not further enhance the inhibitory effect on TOV112D cells (*p* > 0.05), suggesting that a combinatorial therapy may not exert an additive or synergistic effect in this context. However, the combination of MB-50 and METABLOC did moderately affect the proliferation of ARPE-19 cells, indicating some impact on normal cell viability. Furthermore, the addition of carboplatin to MB-50 exhibited a slight impact on TOV112D proliferation compared to MB-50 alone, but this effect was not significant (*p* > 0.05). This suggests a potential additive effect of carboplatin in combination with methylene blue, albeit to a limited extent. However, the MB-50 treatment significantly decreased TOV112D cell proliferation when compared to carboplatin treatment alone (*p* < 0.01), further emphasizing the potential of methylene blue as an adjuvant therapeutic approach for platinum-resistant ovarian cancer. Conversely, carboplatin only marginally affected the proliferation of ARPE-19 cells, indicating a differential response to the treatment between the cancerous and normal cell lines. Overall, these findings support the notion that methylene blue metabolic therapy holds promise for effectively inhibiting the proliferation of ovarian cancer cells.

### 3.2. Impact of Metabolic Therapy on Oxygen Consumption Rate in Normal and Tumor Cell Lines

**Figure 2:**
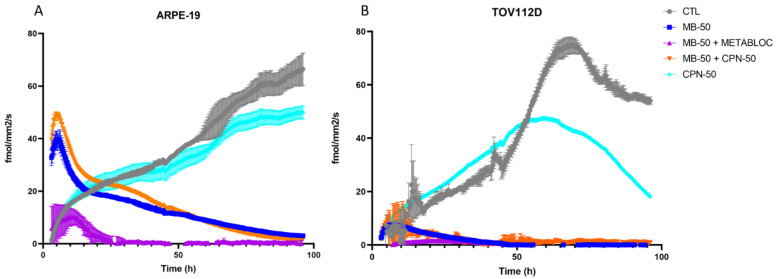
Monitoring of oxygen consumption rate in normal and tumor cell lines under metabolic treatments. The oxygen consumption rate (OCR) was monitored in the normal ARPE-19 cell line and the TOV112D tumor cell line following treatments with methylene blue (MB), METABLOC, or carboplatin, as well as combinations of MB + METABLOC or MB + carboplatin. (**A**,**B**) Initial presence of 50 μM MB (MB-50) in the culture conditions stimulated OCR in both cell lines. However, the presence of METABLOC appeared to mitigate the effect of MB-50 on OCR in both cell lines. (**A**) In ARPE-19, OCR progressively decreased under the influence of MB-50, while CPN-50 condition showed poor effects. (**B**) Carboplatin alone affected TOV112D OCR, with a significant decline initiated at 45 h.

Based on the existing literature, which highlights MB’s preferential localization within mitochondria [32], we sought to investigate MB’s impact on cellular respiration. The results presented in Figure 2 show the effect of the metabolic therapy on the oxygen consumption rate (OCR) of both the normal ARPE-19 and tumor TOV112D cell lines after the treatment with MB, METABLOC, or carboplatin, as well as the combinations of MB + METABLOC or MB + carboplatin. Interestingly, the initial presence of 50 μM MB (MB-50) in the culture conditions stimulated the OCR in both cell lines, suggesting a potential role of MB in enhancing cellular respiration. However, the presence of METABLOC appeared to attenuate the stimulatory effect of MB-50 on the OCR in both cell lines, indicating a modulatory role of METABLOC in the mitochondrial response to MB. In the normal ARPE-19 cell line, the OCR progressively decreased under the influence of MB-50, indicating a potential impact on mitochondrial function. In contrast, the effect of carboplatin alone on the TOV112D OCR was more pronounced, with a significant decline observed after 45 h of treatment. These findings highlight the differential effects of metabolic therapy on the OCR in normal and tumor cell lines, suggesting that the metabolic alterations induced by MB and carboplatin may play a crucial role in cellular respiration and energy metabolism.

### 3.3. Evaluation of Mitochondrial Membrane Potential and Respiratory Complex Gene Expression under Metabolic Therapy

**Figure 3:**
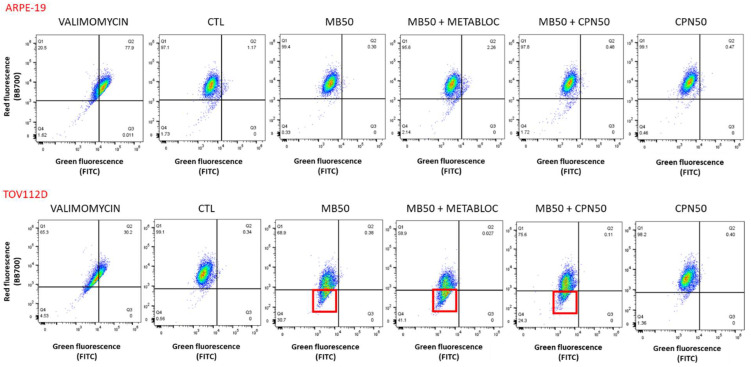
Mitochondrial membrane potential assessment using JC-1 probe in flow cytometry. Valinomycin was used as a positive control for inducing mitochondrial depolarization. The mitochondrial membrane potential of ARPE-19 did not appear to be affected by MB-50 treatment over the short period of 4 h. Under all conditions, both cell populations exhibited polarized mitochondria. However, for the TOV112D cancer cell line, treatment conditions involving methylene blue (MB-50, MB-50 + METABLOC, and MB-50 + CPN-50) showed cell populations in which mitochondria were neither polarized nor depolarized (30.7%, 41.1%, and 24.3%, respectively, red boxes). This phenomenon seems specific to TOV112D ovarian cancer cells.

**Figure 4:**
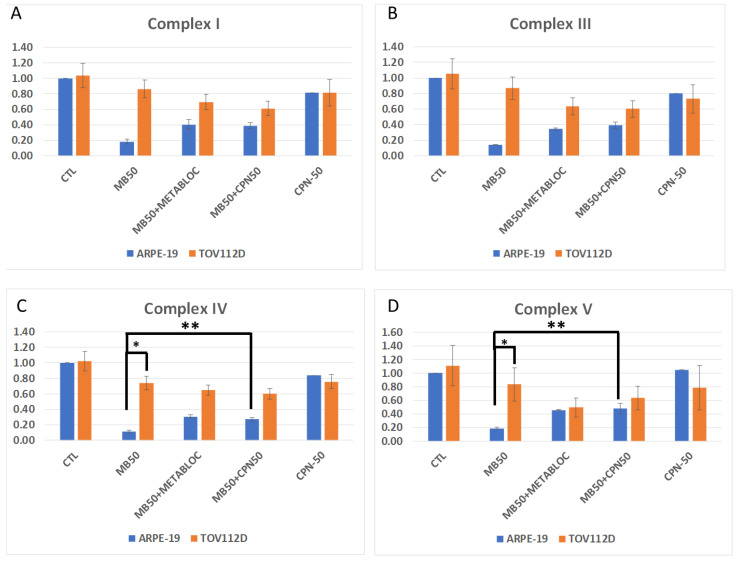
Quantitative PCR of mitochondrial respiratory complex gene. Metabolic treatments influenced the expression of genes encoding protein complexes, including complexes I, III, IV, and V or ATP synthase. (**A**–**D**) Quantitative PCR (qPCR) results revealed similar dynamics of inhibition for all four complexes under the same treatment condition. The normal ARPE-19 cell line was overall more affected than the TOV112D cancer cell line. Particularly, MB-50 treatment strongly suppressed the expression of respiratory complex genes (CI, CIII, CIV, and CV) in the ARPE-19 line (0.18 ± 0.03, 0.14 ± 0.01, 0.11 ± 0.02, and 0.19 ± 0.02, respectively). (**C**) The gene encoding complex IV, responsible for oxygen reduction, was notably repressed in the normal cell line. These findings highlight significant differences in mitochondrial gene expression response to methylene blue between ARPE-19 and TOV112D, with a strong inhibitory effect on ARPE-19. The significant notations are * (*p* ≤ 0.05) and ** (*p* ≤ 0.01).

Figure 3 and Figure 4 provide valuable insights into the evaluation of the mitochondrial membrane potential and the expression of respiratory complex genes in response to metabolic therapy, specifically focusing on the effects of the methylene blue (MB) treatment. Figure 3 depicts the assessment of the mitochondrial membrane potential using the JC-1 probe and flow cytometry in both the TOV112D cancer line and the normal ARPE-19 cell line. Interestingly, over a short period of 4 h, the mitochondrial membrane potential of the ARPE-19 cells did not appear to be affected by the MB-50 treatment. In contrast, the TOV112D cancer line exhibited a distinct response, where the treatment conditions involving methylene blue (MB-50, MB-50 + METABLOC, and MB-50 + CPN-50) resulted in cell populations with mitochondria that were neither polarized nor depolarized. This phenomenon, observed in 30.7%, 41.1%, and 24.3% of the cells, respectively, appears to be specific to TOV112D ovarian cancer cells. These findings suggest that the impact of methylene blue on the mitochondrial membrane potential varies between normal and cancerous cell lines, indicating potential differences in the mitochondrial function and the response to metabolic therapy. Figure 4 focuses on the effects of metabolic therapy on the expression of genes encoding mitochondrial respiratory complexes, including complexes I, III, IV, and V (ATP synthase). The results obtained from quantitative PCR (qPCR) revealed similar dynamics of inhibition for all four complexes under the same treatment condition. Interestingly, the normal ARPE-19 cell line exhibited a more pronounced response compared to the TOV112D cancer cell line. Specifically, the MB-50 treatment strongly suppressed the expression of respiratory complex genes (CI, CIII, CIV, and CV) in the ARPE-19 line, indicating a significant impact on mitochondrial gene expression. Notably, the gene encoding complex IV, responsible for oxygen reduction, was significantly repressed by the MB-50 condition in ARPE-19 (0.11 ± 0.02), compared to 0.74 ± 0.09 in TOV112D (*p* ≤ 0.05). These findings highlight differences in the mitochondrial gene expression response to methylene blue between the ARPE-19 and TOV112D cell lines, with a strong inhibitory effect observed in the normal cell line. This discrepancy suggests that the mitochondria of cancer cells may exhibit a lower sensitivity or altered responsiveness to methylene blue compared to normal cells.

### 3.4. The Effect of Metabolic Therapy on Tumor Growth in Mice

**Figure 5:**
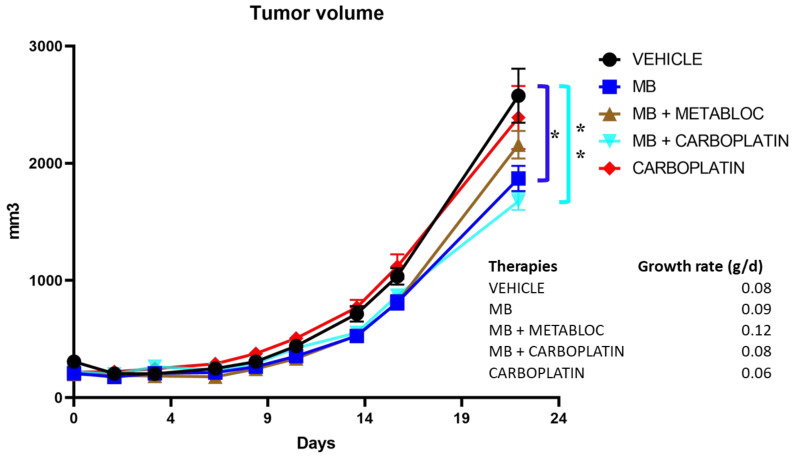
Monitoring tumor progression in mice under metabolic therapies. Tumor progression was monitored in five groups of mice implanted subcutaneously with carboplatin-resistant TOV112D ovarian cancer cell line. The mice were divided into five groups and subjected to distinct treatments for a duration of 22 days: methylene blue (MB), MB in combination with hydroxycitrate and lipoic acid (MB + METABLOC), MB combined with carboplatin (MB + CARBOPLATIN), and carboplatin alone as the conventional treatment. Tumor growth was observed in all groups. However, metabolic therapy (MB alone or combined with carboplatin) demonstrated superior tumor suppression compared to carboplatin treatment. The combination of MB and carboplatin (MB + CARBOPLATIN) displayed a modest enhancement in tumor response compared to MB alone, although the difference was not statistically significant (*p* > 0.05). The respective mouse weight increase (growth rate) in each group showed that metabolic therapies had no major effect on their weight. Note a small weight gain in MB + METABLOC-treated mice. The significant notations are * (*p* ≤ 0.05) and ** (*p* ≤ 0.01).

In view of the positive effects of metabolic therapy in vitro, a preclinical trial was carried out to assess the potential of methylene blue to reduce tumor growth in vivo. We evaluated the tumor growth in a mouse model implanted subcutaneously with carboplatin-resistant TOV112D ovarian cancer cells (Figure 5). The study investigated the efficacy of different treatment regimens, including methylene blue (MB) alone, MB in combination with METABLOC (MB + METABLOC), MB combined with carboplatin (MB + carboplatin), and carboplatin alone as the conventional treatment. The initial tumor volume ranged from 200 to 300 mm^3^ for the mice in each group. Over a duration of 22 days, tumor volume increases were observed in all groups, indicating the aggressive nature of the carboplatin-resistant ovarian cancer model, TOV112D. However, our findings showed a significant decrease in tumor growth in mice receiving the MB-mediated metabolic therapy, either MB alone (1870 ± 108 mm^3^) (*p* ≤ 0.05) or MB in combination with METABLOC (2159 ± 118 mm^3^), compared to the carboplatin treatment (2391 ± 270 mm^3^) or the CTL condition (2576 ± 232 mm^3^). Thus, the metabolic therapies displayed an ability to inhibit tumor progression, suggesting their potential as effective adjuvant treatments for carboplatin-resistant ovarian cancer. Interestingly, the combination of MB and carboplatin (MB + carboplatin) exhibited a modest enhancement in tumor response compared to MB alone, although the difference was not statistically significant (1676 ± 74 mm^3^) (*p* > 0.05). This observation raises the possibility of a synergistic effect between metabolic therapy and carboplatin, warranting further investigation. In addition, no apparent toxicity was observed after these metabolic therapies and no mouse weight loss was detected, as supported by the mouse growth rates (Figure 5). These results support the notion that metabolic therapy holds promise as a viable approach for suppressing tumor growth in carboplatin-resistant ovarian cancer, offering a potential avenue for improving the treatment outcomes in this challenging disease.

## 4. Discussion

The anticancer effect of methylene blue has been known for over a century. In 1893, Louis Rambaud published data on a series of end-stage patients who responded to a high-dose treatment of methylene blue. This was confirmed by Pursell in cancer treatments on dogs [33]. The results presented in this study demonstrate the potential of methylene blue (MB) metabolic therapy as an effective treatment approach for ovarian cancer. The findings reveal the differential sensitivity of the TOV112D ovarian cancer cell line and the normal cell line ARPE-19 to the metabolic treatments employed in this study. The stronger reduction in proliferation observed in TOV112D cells compared to ARPE-19 cells in response to the MB-50 treatment highlights the potential of methylene blue as a therapeutic agent that specifically targets ovarian cancer cells (Figure 1). This finding is particularly interesting considering the challenge of treating platinum-resistant ovarian cancer, as the TOV112D cells exhibited an increased resistance to the carboplatin treatment alone. These results suggest that methylene blue could serve as an additional therapeutic approach for patients with platinum-resistant ovarian cancer. However, the combination therapy of MB-50 and METABLOC showed a moderate impact on the proliferation of ARPE-19 cells, indicating some effect on the viability of this normal cell line, but considering that there was no weight loss in the mice treated with these metabolic therapies (MB and METABLOC), this suggests that a therapeutic index might be achieved. This raises the need for further investigation into the potential side effects or cytotoxicity of the combined treatment on normal cells. Additionally, the addition of carboplatin to MB-50 exhibited a slight impact on the TOV112D proliferation compared to MB-50 alone, suggesting a potential additive effect of carboplatin in combination with methylene blue, albeit to a limited extent. The modest enhancement of the in vivo tumor response observed to the combination of MB and carboplatin (MB + carboplatin) raises the possibility of a synergistic effect between the metabolic therapy and carboplatin (Figure 5). This preclinical study conducted in a mouse model implanted with carboplatin-resistant TOV112D ovarian cancer tumors provides evidence regarding the efficacy of metabolic therapy in reducing tumor growth (Figure 5). A significant decrease in tumor growth was observed in response to MB-mediated metabolic therapy, either MB alone or MB in combination with METABLOC, compared to the carboplatin treatment, suggesting the potential of metabolic therapies as alternative treatments for carboplatin-resistant ovarian cancer. However, the observation that methylene blue does not reverse tumor growth, either alone or in combination with carboplatin, suggests that the in vivo assimilation of MB may pose a limitation for therapeutic recommendations. Further investigation into the assimilation rate of MB is needed. These findings support the notion that metabolic therapy holds promise as a viable approach for suppressing tumor growth by specifically targeting mitochondrial energetics (Figure 2 and Figure 3). The impact of metabolic therapy on the oxygen consumption rate (OCR) in normal and tumor cell lines sheds light on the potential mechanisms underlying mitochondrial dynamics. The stimulatory effect of MB-50 on the OCR in both cell lines, attenuated by the presence of METABLOC, suggests a modulatory role of METABLOC in the mitochondrial saturation response to increased carbon flux [28,31]. The progressive decrease in the OCR observed in the normal ARPE-19 cell line under the influence of MB-50 indicates a potential impact on mitochondrial horsepower [34]. Conversely, the effect of carboplatin alone on the TOV112D OCR was more pronounced, emphasizing the differential effects of metabolic therapy on the OCR in normal and tumor cell lines. These findings suggest that the metabolic alterations induced by MB and carboplatin may play a crucial role in cellular respiration and energy metabolism. The evaluation of the mitochondrial membrane potential and the expression of respiratory complex genes provides insights into the response of normal and cancerous cell lines to metabolic therapy. However, it would be highly relevant to identify the metabolic pathways as well as the signaling pathways involved in the inhibition of cancer cell growth by methylene blue. In this study, we targeted specific parameters of the mitochondria to provide insights. The distinct response of the TOV112D cancer cells to the MB treatment, resulting in an unmarked cell population according to JC-1, indicates potential differences in the mitochondrial function between the normal cell line ARPE-19 and the ovarian cancer cell line TOV112D. Moreover, the stronger inhibitory effect of the MB-50 treatment on the expression of respiratory complex genes in the normal ARPE-19 cell line suggests a higher MB overflow into the mitochondria compared to TOV112D. These differences in the mitochondrial gene expression response to methylene blue between normal and cancer cell lines highlight the potential altered responsiveness or redox status of the mitochondria of cancer cells [22].

## 5. Conclusions

In conclusion, this study provides crucial insights into the potential of methylene blue-guided metabolic therapy for treating ovarian cancer. The results underscore the promise of metabolic therapy in reducing tumor growth by addressing the mitochondria redox potential, akin to its role as an oxidative agent in bacterial diseases. However, there is a pressing need for further investigations into the specific impact of methylene blue on apoptotic signaling and metabolic pathways within cancer cells. These molecular mechanisms must be thoroughly elucidated to optimize the application of metabolic therapy and enhance the treatment outcomes, particularly in the context of chemoresistant ovarian cancers. The potential development of targeted metabolic strategies based on these findings opens avenues for significant advancements in cancer treatment by remotely modulating mitochondrial activity, inverting their embryonic-like redox potential [35,36], and inducing controlled apoptosis, reminiscent of the principles seen in photodynamic therapy [32,37].

## Data Availability

The data presented in this study are available in this article.
